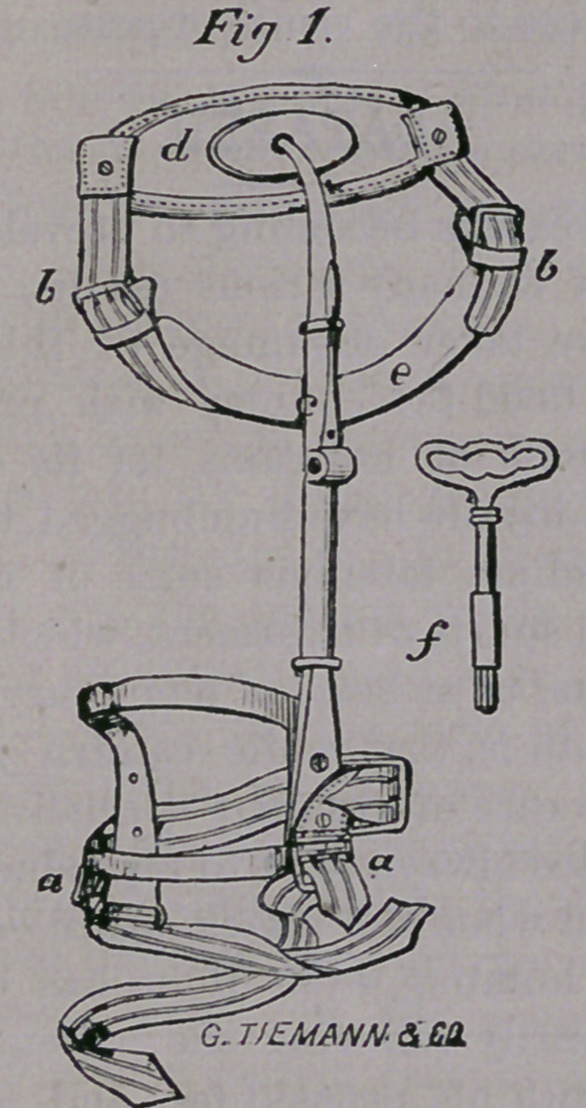# Morbus Coxarius, or Hip Joint Disease

**Published:** 1874-07

**Authors:** 


### MORBUS COXARIUS, OR HIP JOINT DISEASE.

This is a difficulty so little understood by parents, and for this reason, doubtless, neglected, until the child has reached that stage in the disease which renders a cure problematical, and a cripple certain. In view of this fact a description of the malady, with a statement of its stages and results, might be instrumental in saving some suffering little one from the terrible ravages of this distressing complaint.

The disease is usually confined to those frail and anæmic children, blessed with bright intelligent faces, but thin in flesh and weak in strength. The first noticeable symptom is a limp, most observed in the morning, and passing off during the day. Some days may pass without the child's limping or complaining of the least discomfort, but returns again after a lengthened play or a day of unusual fatigue.

After a while, pain is complained of and is generally referred to the knee, although pressure or examination of that point will not increase the trouble, and no pain will be felt until you move the hip joint. Taking the child's limb and bending it upon his body in such a manner as to bring great strain upon the hip joint, will make him cry with pain. This is the *first stage* of the disease, and one in which the difficulty is easily remedied.

An apparatus, as here figured, is immediately applied to the limb, which has for its object the removal of pressure upon the joint.

*Fig 1.*

This apparatus consists of three portions, the upper one made of steel, in which there is a ball-and-socket joint, capable of movement in every direction, and attached to a soft pad (d) which accurately fits the back, between the hip bones. From this steel brace is suspended a perineal band (e), which passes between the limbs and is adjusted by strong webbing and buckles (b b). The middle portion consists of a bar which is lengthened or shortened by means of a ratchet, worked by the key (f). The bands at the lower portion, (a a) embrace the thigh just above the knee. When the bar is elongated by means of the ratchet, the limb is extended, drawing the head of the thigh bone gently from contact with the socket, and preventing further inflammation in the joint while the healing process is going on.

As intimated, this apparatus should be applied early, else will the disease progress to the *second stage*, characterised, by a seeming elongation of the limb, with a slight flexion, or drawing up of the knee, toward the body. The pelvis or hip bones are tilted toward the affected side, and the fleshy portion immediately in the rear of the hip-joint, becomes flattened and depressed. The child now steps upon his toes, and complains of much pain, particularly at night, often awakening from a deep slumber with a shriek from the intense pain which suddenly attacks him.

Even at this stage, suitable apparatus can be applied, and treatment adopted that often results in a cure, otherwise the disease progresses to the third stage, when abscesses from in and about the hip-joint, and fistulous tracts are formed communicating often with the cavity of the abdomen and with the bowels. In the case of a child which recently came under our observation, the abscess communicated by a fistulous opening with the intestines, through which the contents of the bowels were daily discharged. The condition of this little boy was such that tearful sympathy would be drawn from the stoutest hearts, in witnessing his patient yet terrible sufferings. At this stage of the disease no help remains save that obtained from a surgical operation. This consists in removing the head of the diseased bone, or such other portions as may be involved in the difficulty. The little sufferer is then placed in wire-breeches, and awaits the healing of the wound. Usually all pain is at once relieved through this operation and the patient makes a reasonably rapid recovery. Shortening of the limb occurs in proportion to the amount of bone the surgeon finds it necessary to remove. This defect is relieved by mechanical appliances of various kinds, usually consisting in attaching a cork sole or steel frame to the shoe, to give the required length to the limb.

If parents will but see to such diseases early, seeking good surgical advice as soon as the symptoms first given present themselves they will not only save much suffering to themselves but also untold agony to their innocent and helpless children.

## Figures and Tables

**Fig 1. f1:**